# Discussion themes and emotional expressions on Chinese social media regarding AI virtual companions: a topic modeling and sentiment analysis study

**DOI:** 10.3389/fpsyg.2026.1841223

**Published:** 2026-06-03

**Authors:** Yue Sun, Jun Liu, Ting Liu, Zhengqi Wei, Huajie Shen

**Affiliations:** 1 School of Design, Fujian University of Technology, Fuzhou, China; 2 College of Art, Cheongju University, Cheongju, Republic of Korea

**Keywords:** AI virtual companions, Chinese social media, human-AI relationships, sentiment analysis, topic modeling, user experience

## Abstract

**Background:**

As AI virtual companions become part of everyday life, they have attracted growing public and scholarly attention. However, evidence remains limited on how users understand, express, and evaluate these systems in real online contexts. Existing studies have relied largely on surveys or experiments, with relatively few examining discussion themes and emotional structures around AI virtual companions in authentic online settings. Against this background, this study examines discussion patterns surrounding AI virtual companions in Chinese social media and the structure of researcher-constructed higher-order experiential categories reflected in users’ comments.

**Methods:**

A total of 20,807 comments from 168 relevant Xiaohongshu posts were collected as raw data. After text cleaning, BERTopic was used for initial topic modeling, followed by manual review to refine and consolidate the topics. Sentiment analysis was conducted by fine-tuning a Chinese RoBERTa model on a manually labeled corpus.

**Results:**

The study identified 29 topics, further integrated into seven higher-order experiential categories. The most frequently discussed topics included Perceptions of Monetization and Platform Mechanisms, Relationship Memory and Continuity, Virtual Romance Experience, and Controversies Over Respect in Interaction, indicating that user discussions extended beyond simple functional evaluation. Significant differences were found across topics and higher-order experiential categories in sentiment distribution and sentiment scores. Emotional Gains and Losses, Positive Emotions, and Virtual Romance Experience showed more positive tendencies, whereas Usage Restrictions, Originality Disputes, Privacy Risks, and Discussions of Boundary Violations and Sensitive Content showed more negative tendencies. At the higher-order experiential category level, Virtual Intimacy and Boundary Negotiation and Emotional Engagement and Regulation showed more positive emotional tendencies overall, whereas Risk Perception and Ethical Boundaries and Platform Functionality and Monetization showed more negative tendencies.

**Conclusion:**

Online discussions of AI virtual companions are no longer limited to technical functions or use evaluations, but increasingly involve relationship construction, emotional experience, and boundary negotiation. Users’ emotional expressions are shaped by specific discussion contexts and relational meanings. By integrating topic modeling with sentiment analysis, this study provides structured empirical evidence for understanding user experiences with AI virtual companions in Chinese social media and offers implications for future research and design on relational AI.

## 1 Introduction

In recent years, with the development of artificial intelligence and natural language processing technologies, systems capable of sustained conversation and emotional responsiveness have gradually become integrated into everyday life (Chan and Chou, 1997). Among them, AI virtual companions, as an emerging form of human–AI interaction, have attracted widespread attention because they can provide emotional communication, social companionship, and everyday support (Chaturvedi et al., 2023). Unlike traditional intelligent assistants that mainly focus on task execution and information support, AI virtual companions place greater emphasis on relational interaction and emotional responsiveness. As a result, they are, to some extent, experienced by users as “digital social objects” with whom ongoing communication is possible (Kouros and Papa, 2024; Zhou et al., 2020).

Existing research has examined the influence of AI companion systems on user experiences from multiple perspectives (Colby et al., 1971). For example, anthropomorphic features are believed to enhance users’ emotional engagement and interaction involvement while also facilitating the development of trust and social presence (Chou et al., 2025; Liu et al., 2025). At the same time, related studies have explored users’ emotional experiences during interaction, including feelings of companionship, identification, and emotional support (Zhang et al., 2025). Meanwhile, some studies have begun to address the potential psychological risks associated with the use of AI companion systems, suggesting that some users may experience relatively high levels of loneliness and may develop strong dependence on virtual relationships through continued interaction (Wijesundara and Rathnayake, 2024). Together, these studies have provided an important foundation for understanding user experiences with AI companion systems.

Despite this progress, several limitations remain in the existing literature (Liu et al., 2025). First, most studies rely on survey or experimental data. Although these approaches are useful for testing relationships among variables, they are limited in their ability to capture users’ natural expressions in real interaction contexts (Liu et al., 2025; Mingxi and Zhifeng, 2025; Ocal, 2023, 2024; Zhang et al., 2025). In contrast, social media data are more spontaneous and contextualized and can more directly reflect public views and experiences (Öcal et al., 2021). However, in the context of AI virtual companions, existing research has still rarely made systematic use of social media data to analyze the specific content of user discussions. In particular, in the Chinese social media context, relevant studies have not yet adequately revealed the thematic structure that emerges in discussions of AI virtual companions.

Second, although prior studies have examined psychological experiences associated with the use of AI companion systems, including companionship, attachment, well-being, emotional support, and potential dependence (Liu et al., 2025; Wijesundara and Rathnayake, 2024; Zhang et al., 2025), systematic investigation of differences in emotional expression across discussion themes remains relatively limited. Because AI virtual companions are not a single functional technology object, user discussions often involve multiple contexts at once, including relational continuity, emotional investment, real-life impacts, platform governance, and ethical risks. Therefore, identifying only “what is being discussed” is not sufficient to fully understand user experience. Further comparison of the sentiment distribution associated with different discussion themes can help reveal how users assign different relational meanings and value judgments to AI across differentiated interaction contexts.

Third, existing research has mainly focused on single-variable relationships or specific types of experience and has paid limited attention to whether dispersed discussion themes can be further integrated into higher-order experiential categories. Therefore, user experience with AI virtual companions needs to be examined not only at the topic level, but also in terms of whether these topics can be organized into higher-level structures, so as to more systematically understand the possible organization of user experience.

From a psychological perspective, users’ emotional responses often arise from their cognitive appraisal of specific situations (McEachrane, 2009; Moors et al., 2013). To some extent, different discussion themes correspond to different interaction contexts, and users’ emotional expressions within these contexts reflect their subjective judgments about situational meaning. On this basis, topics with similar semantics or related concerns may be further grouped into higher-order experiential categories. Accordingly, this study uses Chinese social media comments as the research data and conducts an exploratory analysis of user discussions about AI virtual companions. It focuses on topic content, topic-level emotional distributions, and the possible organization of higher-order experiential categories. Specifically, this study addresses the following research questions:

*RQ1*: What themes do users mainly discuss?

*RQ2*: What distributional patterns characterize emotional expressions across different themes?

*RQ3*: Based on the identified topic content, can these topics be further integrated into higher-order experiential categories?

Methodologically, this study draws on Chinese social media comment data and combines BERTopic topic modeling with sentiment analysis to systematically examine online discussions of AI virtual companions. Specifically, the study first identifies the main topics in user discussions and then compares differences in emotional distributions across topics. On this basis, related topics are further integrated to explore the possible organization of higher-order experiential categories. The overall emotional patterns of these experiential categories are then described. By integrating these two methods, this study is able not only to show what users discuss, but also to further reveal the emotional differences associated with different kinds of discussion content.

The contributions of this study are threefold. First, at the empirical level, this study systematically identifies discussion topics about AI virtual companions in an authentic social media context and attempts to group them into higher-order experiential categories. In doing so, it addresses the limited attention that prior research has paid to natural online expressions. Second, at the methodological level, this study combines topic modeling and sentiment analysis to compare emotional expression across topics and further examine overall emotional patterns at the experiential-category level, providing a useful example of text mining methods in psychology and human–AI interaction research. Third, at the theoretical level, this study does not directly test existing theories of relationships or emotion. Instead, it provides empirical clues from the Chinese social media context for understanding the relational meanings, emotional differences, and possible organization of experiences reflected in discussions of AI virtual companions.

## 2 Literature review

### 2.1 AI virtual companions and the evolution of human–AI relationships

With the rapid development of artificial intelligence and natural language processing technologies, AI virtual companions capable of sustained interaction with users have gradually entered public view (Liu et al., 2025). Unlike traditional intelligent systems oriented toward task execution, these systems provide emotional communication and social companionship through natural language conversation and emotional feedback (Schuetzler et al., 2020; Shawar and Atwell, 2007). Against the backdrop of increasing numbers of people living alone and growing demand for companionship in real life, AI virtual companions have gradually become an important topic of research in human–computer interaction and affective computing (Berridge et al., 2023; Gasteiger et al., 2022; Rook, 1987).

From a developmental perspective, human–AI relationships have evolved from instrumental use to social interaction, and then to affective relationships (Kropf, 2025; Liang and Lu, 2025). Early dialogue systems mainly relied on rule-based mechanisms and were limited in their ability to support sustained interaction and complex emotional communication (Colby et al., 1971; Wallace, 2009; Weizenbaum, 1966). With advances in natural language processing and machine learning, especially the application of large language models, the new generation of AI virtual companion systems has developed stronger semantic understanding and generative capabilities, making human–AI interaction more natural and continuous (Kouros and Papa, 2024). This technological progress has driven virtual companion systems to shift from being mere information-processing tools to becoming interactive objects with social attributes, thereby giving them an increasingly important place in human–computer interaction and affective computing research (Merrill Jr et al., 2022; Zhang and Li, 2025).

On this basis, researchers have begun to pay greater attention to users’ psychological experiences in interactions with AI virtual companions (Alabed et al., 2024). Existing studies suggest that anthropomorphic features can enhance users’ emotional engagement and increase feelings of companionship and subjective well-being (Hu et al., 2025; Xie et al., 2023). At the same time, AI virtual companions may be regarded to some extent as nonjudgmental listeners, helping users express emotions and relieve psychological stress (Kouros and Papa, 2024). Further studies have shown that, through sustained interaction, users may gradually assign AI relational meaning that goes beyond its functional attributes and may display tendencies toward intimacy, dependence, and ongoing attention in the course of interaction (Liu et al., 2025). These studies reveal, at the level of psychological experience, how users develop emotional engagement and relational connection through interaction with AI systems (Gong and Zhang, 2025; Xie and Wang, 2024), and they also provide an important foundation for understanding relational imagination and interactive experiences in discussions related to AI virtual companions.

However, existing research on AI virtual companions has relied primarily on interviews, surveys, experiments, or mixed methods, with a main focus on attachment, relationship development, psychological consequences, or interface preferences. As a result, it has been difficult to fully capture users’ natural expressions in real-world contexts (Liu et al., 2025; Mingxi and Zhifeng, 2025; Zhang et al., 2025). With the development of social media platforms, user-generated content has opened a new perspective for understanding how the public views AI virtual companions (Zhen and Zhang, 2025). By comparison, studies that draw on large-scale public social media texts and combine topic modeling with sentiment analysis to examine user discussion content and its higher-order emotional structure remain relatively limited, especially in the Chinese social media context. Therefore, further research is still needed to examine how users discuss AI virtual companions in real contexts and how these discussions reflect different relational concerns and emotional expressions. To present more clearly the main study objects, methodological approaches, research focuses, and limitations of existing empirical studies on AI virtual companions, this study provides a systematic review of representative prior work (see Table 1).

**Table 1:** Summary of empirical studies on AI virtual companions.

Authors	Study object/context	Method and sample	Research focus	Relation to the present study/remaining limitation
Merrill Jr et al., 2022	AI companions for lonely individuals	Online experiment + survey	Social presence, warmth, usefulness, recommendation	Focuses on perception and acceptance, but remains primarily centered on scale-based variables and does not analyze naturally occurring social media discussions or their emotional structure
Kouros and Papa (2024)	Replika users	User interviews + digital ethnography	Emotional attachment, self-expression, identity exploration	Provides in-depth qualitative evidence, but the sample is relatively small and lacks large-scale analysis of public discussions
Xie et al. (2023)	Replika/social chatbot users	Mixed methods	Loneliness, trust, personification, relationship development, psychological dependence	Examines relationship development and dependence, but still relies mainly on interviews and scale-based variables rather than publicly occurring social media discourse
Xie and Wang (2024)	Virtual companionship use among Chinese university students	Two-wave longitudinal survey (*N* = 618)	Social anxiety, emotional expression, mindfulness	Focuses on psychological outcomes, but remains mainly based on scale variables and does not reveal the thematic structure of user discussions
Zhang and Li (2025)	Human–AI love among Chinese women	Semi-structured interviews (*N* = 14)	Formation and maintenance of intimate relationships, over-immersion, boundaries between virtual and real life	Provides evidence on intimate relationship experiences in the Chinese context, but lacks large-scale analysis of public discussions
Liu et al. (2025)	Long-term Chinese users of AI virtual companion apps	Interviews (*n* = 10) + survey (*N* = 612)	Attachment, loneliness, wellbeing, self-concept clarity, real-life social participation	Focuses on attachment pathways among long-term users, but remains mainly based on scale variables and lacks large-scale analysis of public discussions

### 2.2 Theoretical explanations of human–AI emotional relationships and emotional responses

To understand users’ relational expressions and emotional responses in discussions of AI virtual companions, this study introduces parasocial interaction theory, attachment theory, and appraisal theory from a psychological perspective as theoretical references for interpreting the findings. It should be noted that these theories are not used here as an analytical framework that directly guides the formulation of the research questions, analytical decisions, or formal theory testing. Rather, they are adopted as interpretive perspectives for understanding the thematic content, emotional differences, and their possible psychological meanings identified in real social media contexts. Taken together, these three theories provide support for understanding differences in user expression across different discussion themes from the three levels of relationship formation, relationship deepening, and emotion generation (Jarzyna, 2021; McEachrane, 2009; Yang and Oshio, 2025).

First, parasocial interaction theory provides an important basis for understanding how individuals form psychological connections with non-real entities (Horton and Richard Wohl, 1956; Jarzyna, 2021). This theory suggests that individuals can develop interpersonal-like psychological connections with virtual characters or other non-real objects in mediated contexts (Liu, 2025). In the context of AI virtual companions, features such as natural language conversation, emotional feedback, and sustained interaction make it easier for users to perceive the system as an entity with “otherness,” thereby gradually forming quasi-social relationships (Jin et al., 2026). Accordingly, AI virtual companions may no longer be viewed only as tools, but may also be experienced as interactive objects with social meaning (Pentina et al., 2023).

Second, attachment theory helps further explain how human–AI relationships move from being formed to being sustained and deepened (Yang and Oshio, 2025). Attachment theory proposes that individuals develop attachments to stable emotional objects through ongoing interaction and seek security and emotional support from them (Heng and Zhang, 2025). In the context of AI virtual companions, users may gradually come to view AI as a dependable emotional object through continued interaction and emotional communication (Hu et al., 2025). In digital settings, this relationship may take the form of digital attachment, meaning that users develop emotional attachment and psychological dependence toward virtual interaction objects (Cundy, 2018). This perspective helps explain users’ expressive tendencies in discussions of virtual romance, emotional dependence, and relationship continuity (Yang and Oshio, 2025; Zhang et al., 2022).

However, focusing only on relationship formation and attachment is still insufficient to explain emotional differences across discussion themes. From the perspective of emotion psychology, emotional responses typically arise from individuals’ cognitive appraisals of specific situations (Moors et al., 2013). Appraisal theory of emotion holds that individuals generate corresponding emotional responses based on their judgments of an event’s meaning, such as its relevance, controllability, or favorability (McEachrane, 2009). In discussions of AI virtual companions, different themes can be understood as different interaction contexts, such as emotional support, relational conflict, ethical risk, or functional restrictions. By shaping individuals’ judgments about situational meaning, these contexts further give rise to differentiated emotional expressions. Therefore, appraisal theory of emotion provides an important explanatory path for understanding differences in sentiment distribution across different discussion themes.

In sum, parasocial interaction theory, attachment theory, and appraisal theory of emotion provide valuable conceptual perspectives for understanding users’ discussion content and sentiment differences regarding AI virtual companions from the three levels of relationship formation, relationship deepening, and emotion generation. It should be noted that this study does not directly test these theories. Rather, they are used as an interpretive framework for understanding the thematic content, higher-order experiential categories, and associated patterns of emotional expression identified in real social media contexts. On this basis, the present study interprets online discussions of AI virtual companions from the perspective of interaction context–relational meaning–emotional expression through exploratory text analysis.

### 2.3 User expression on social media and text mining methods

Building on the theoretical perspectives above, identifying users’ discussion themes and emotional expressions related to AI virtual companions in real contexts requires data sources that reflect natural interaction processes. With the development of social media platforms, user-generated content has gradually become an important source of data for understanding public online discussions and emotional expressions (Suk et al., 2025). Compared with traditional survey or experimental research, social media texts are more spontaneous and contextualized and can more directly present individuals’ experiences, views, and emotional attitudes in natural interaction settings (Suk et al., 2025). As a result, an increasing number of studies have begun to use social media data to analyse public discussion of specific issues, thereby compensating for the limited ecological validity of traditional methods (Fu et al., 2025; Lian et al., 2024; Qi et al., 2024).

From an analytical perspective, understanding user expression on social media usually involves two core questions: what users are discussing and how users express emotion. In text mining, topic modelling is widely used to identify latent semantic structures in texts and thereby reveal the main issues discussed by users (Islam, 2019; Justicia De La Torre et al., 2018). By analysing patterns of word co-occurrence, topic modelling can extract recurring thematic structures from large-scale texts, and it has therefore been widely used in social media and public opinion research (Shu et al., 2024). Early studies mainly adopted probabilistic models such as latent Dirichlet allocation (LDA) (Blei et al., 2003; Egger and Yu, 2022b). However, because these methods rely on word-frequency statistics, they are limited in their ability to capture semantic relationships among words and therefore have certain shortcomings in short-text contexts (Egger and Yu, 2022a; Grootendorst, 2022; Sánchez-Franco and Rey-Moreno, 2022). With advances in natural language processing, topic modelling methods based on semantic embeddings, such as BERTopic, combine pre-trained language models with clustering algorithms to identify semantic similarity in texts more accurately, thereby improving the interpretability and stability of topic identification (Egger et al., 2022; Egger and Yu, 2022a; Ferizaj et al., 2025).

At the same time, sentiment analysis is widely used to identify emotional tendencies in texts and thus characterize users’ emotional expressions (Lin et al., 2026; Wankhade et al., 2022). Early methods mainly relied on sentiment lexicons or rule-based matching (Alkan et al., 2023; Liu et al., 2023), which had limitations when dealing with complex contexts and implicit emotions (Liu et al., 2022). Later, machine learning–based methods improved the accuracy of sentiment recognition by training classification models (Jianzhong et al., 2017; Zhu and Goldberg, 2009). In recent years, deep learning methods based on pre-trained language models such as BERT have been better able to capture contextual semantic information and have shown greater stability and accuracy in sentiment classification tasks (Anggrainingsih et al., 2023; Devlin et al., 2019; Jena, 2020).

As social media data have become more widely used in social science research, some studies have begun to combine topic modelling with sentiment analysis to simultaneously identify the thematic structure of online discussions and the corresponding patterns of emotional expression, thereby offering a more comprehensive account of users’ discussion characteristics and emotional responses in online interaction (Li and Hu, 2025; Lin et al., 2026; Nemes and Kiss, 2021). However, relatively few existing studies have applied this integrated analytical approach to the Chinese social media context related to AI virtual companions.

Accordingly, this study combines topic modelling with sentiment analysis to conduct a systematic analysis of Chinese social media comment data. It aims to identify the major discussion themes surrounding AI virtual companions, compare the distribution of emotional expressions across different themes, and further examine whether these themes can be integrated into higher-order experiential categories. In doing so, the study provides empirical evidence for understanding users’ online discussions and emotional expressions in real interaction contexts.

## 3 Methodology

**Figure 1:**
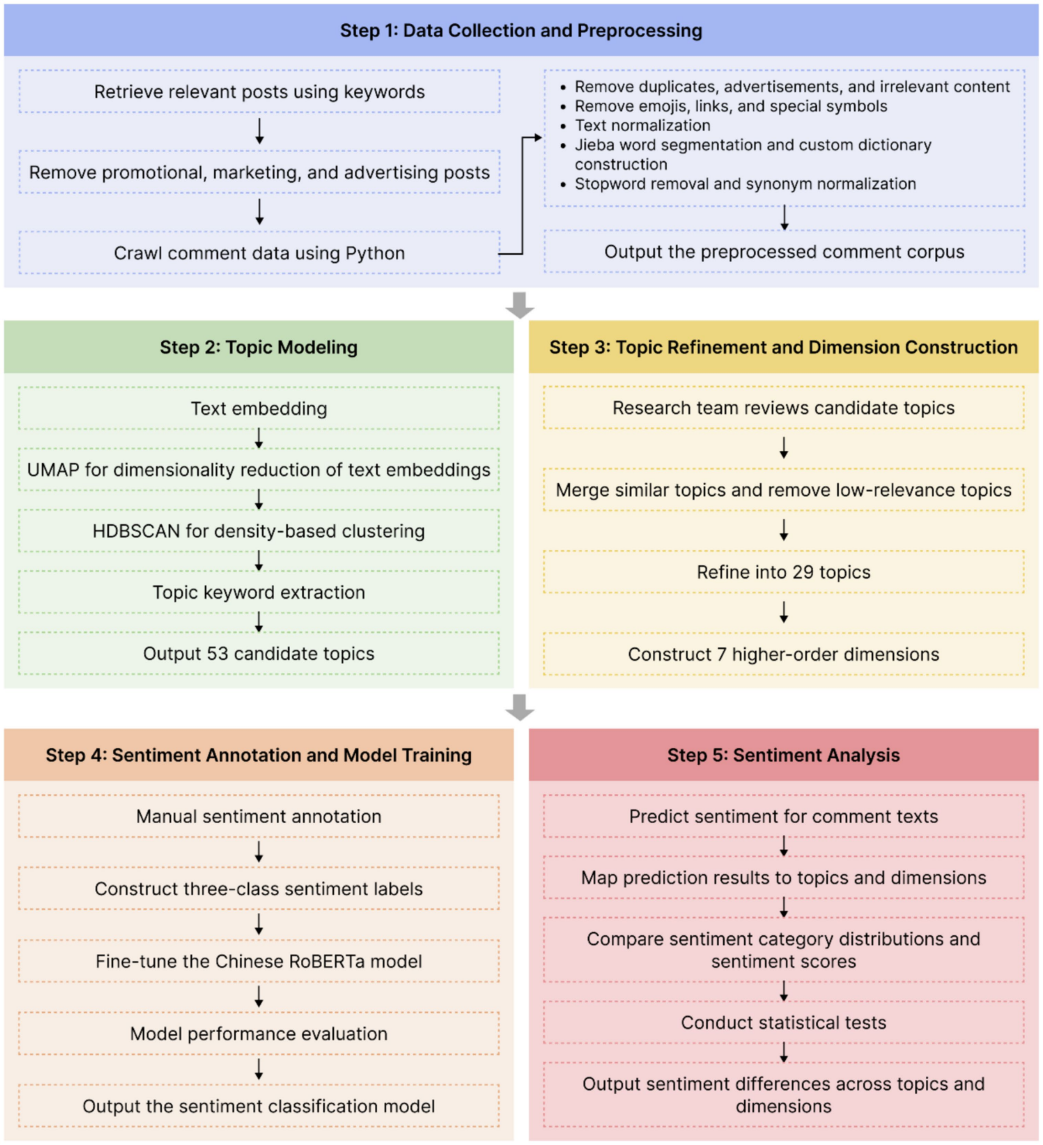
Research workflow.

This study adopted a multi-stage analytical procedure to systematically identify the discussion topics and emotional expressions of social media users regarding AI virtual companions. The overall research design consisted of five steps: data collection and preprocessing, BERTopic-based topic modeling, topic refinement and experiential category construction, sentiment annotation and model training, and statistical analysis of emotions at the topic and experiential-category levels. Figure 1 presents the overall workflow of the study.

### 3.1 Data collection and preprocessing

To reveal users’ discussion themes and emotional expressions related to AI virtual companions in natural usage contexts, this study selected the Chinese social media platform Xiaohongshu as the data source and analyzed relevant posts and their comments. This platform centers on community interaction and experience sharing, where users typically express themselves through personal experiences and emotional reflections (Ji et al., 2025). Compared with general social media platforms that are oriented more toward information dissemination, Xiaohongshu is better suited to capturing users’ ongoing experiences with emotion-oriented AI products and their complex psychological responses (Zhu, 2026). Therefore, it provided text data with strong contextual and interactive features for the present study.

The data were collected using Python-based web scraping techniques. Based on predefined keywords and a specified time range, relevant pages were automatically accessed and parsed to collect posts and comment texts related to the research topic. To improve data relevance and reduce interference from commercial content, a preliminary screening process was conducted during data collection. Posts primarily intended for product promotion, marketing, or advertising were excluded, including those containing standardized promotional language, purchase-oriented links, or repetitive commercial text. A multi-level retrieval strategy was used for keyword selection, including both conceptual terms (e.g., “AI virtual companion,” “AI lover,” and “virtual romance”) and specific product names (e.g., “Xingye,” “Maoxiang,” and “Zhumengdao”), in order to balance data coverage and relevance. On this basis, the researchers further conducted a manual review of the search results and retained only posts and comments that explicitly concerned AI virtual companion–related products, interaction experiences, or usage practices. The data collection period ranged from April 2024 to January 2026. A total of 20,807 user comments from 168 relevant posts were obtained, forming the initial dataset for this study. All data were derived from publicly accessible content and were anonymized in subsequent analyses to comply with academic ethical standards.

To improve the reliability of text analysis and enhance the transparency of corpus screening, the raw comment data were further subjected to systematic preprocessing. A total of 20,807 comments were obtained at the initial stage. First, duplicate texts and obviously irrelevant content, such as advertisements, external links, and non-semantic symbols, were removed. Second, anomalous characters and obvious spelling problems were normalized to improve textual consistency. The comments were then segmented using Jieba, and a custom dictionary was constructed based on the research context to ensure accurate identification of key terms (Zhu, 2026). At the same time, stop-word removal and synonym normalization of semantically similar expressions were performed to reduce semantic redundancy and improve corpus consistency. After text cleaning, segmentation, and filtering of empty texts, 17,358 valid comments were retained. BERTopic was then used for initial topic modeling, in which 8,237 comments were assigned to Topic −1. Because Topic −1 in BERTopic usually represents outlier or noisy texts that do not form stable clusters, these comments were excluded from the subsequent analysis of thematic structure. A sampled inspection showed that these comments were mostly short replies, fragmented expressions with insufficient context, generalized casual remarks, or semantically dispersed content, making it difficult for them to form stable and interpretable discussion themes. After their removal, the remaining 9,121 comments entered the topic refinement stage. During topic refinement, the research team further merged, excluded, and consolidated candidate topics according to semantic consistency, expressive intent, and research relevance. Candidate topics composed mainly of generalized expressions, weakly relevant content, or semantically ambiguous material were excluded from subsequent analysis, along with their corresponding comments. Ultimately, 7,398 comments were retained, forming the final analytical corpus for the subsequent topic analysis, sentiment prediction, and statistical testing. Table 2 reports the number and proportion of comments retained and excluded at each stage, as well as the main rationale for each processing decision.

**Table 2:** Corpus screening process and audit of excluded comments.

Stage	Processing outcome	Number of comments	Proportion of previous stage	Proportion of original corpus	Basic characteristics or reason for exclusion
Initial comment collection	Retained	20,807	—	100.00%	Original comments collected from 168 relevant Xiaohongshu posts
Data cleaning and preprocessing	Excluded	3,449	16.58%	16.58%	Mainly included duplicate texts, advertisements, external links, non-semantic symbols, empty texts, and clearly irrelevant content
Valid comments after cleaning	Retained	17,358	83.42%	83.42%	Entered the initial BERTopic topic modeling stage
BERTopic Topic −1	Excluded	8,237	47.45%	39.59%	Mainly outlier texts that could not form stable topic clusters, including short replies, fragmented expressions lacking sufficient context, generalized casual comments, and semantically dispersed comments
Entered the topic refinement stage	Retained	9,121	52.55%	43.84%	Entered manual review and topic refinement of the 53 candidate topics
Topic refinement stage	Excluded	1,723	18.89%	8.28%	Mainly comments with insufficient semantic coherence, weak research relevance, ambiguous topic boundaries, or difficulty forming stable interpretive units
Final analytical corpus	Retained	7,398	81.11%	35.56%	Used for the final topic analysis, sentiment prediction, and statistical tests

Topic −1 refers to comments that BERTopic could not assign to stable topic clusters. These comments were excluded to improve the semantic clarity and interpretive stability of the topic structure, rather than to selectively remove any particular sentiment tendency or viewpoint type.

It should be noted that the unit of analysis in this study was the comment text rather than the individual user. The study aimed to identify the patterns of public discussion and emotional expression surrounding AI virtual companions on Xiaohongshu, rather than to treat each individual comment as automatically equivalent to a stable user-level attitude or psychological state. Because the data were drawn from publicly accessible platform pages, and no user-level identifiers that would allow stable tracking of individual identities were retained during data collection and anonymization, this study did not perform precise user-level identification or matching of repeated commenters. Accordingly, the study does not attempt to make inferences at the user level, but instead analyzes comments as contextualized units of expression. At the same time, given Xiaohongshu’s specific platform culture, user composition, and expressive style, the findings of this study mainly reflect the characteristics of public discussion surrounding AI virtual companions in this particular platform context, and caution is therefore needed in generalizing them beyond this setting.

### 3.2 Topic modeling with BERTopic

To identify the core issues discussed by users regarding AI virtual companions in the Chinese social media context, this study employed BERTopic for topic modelling (Wang et al., 2024). This method combines semantic embeddings, UMAP-based dimensionality reduction, HDBSCAN-based density clustering, and c-TF-IDF-based keyword extraction, making it well suited to handling the short texts, semantic dispersion, and diverse expressions commonly found in social media comments (Janssens et al., 2025; Ma et al., 2025; McInnes et al., 2018).

Specifically, the study first used bert-base-chinese to generate vector representations of the comment texts. Next, UMAP was applied to reduce the dimensionality of the text embeddings, with the following parameter settings: n_neighbors = 15, n_components = 5, min_dist = 0.0, and metric = cosine. In addition, random_state = 42 was specified to improve the stability and reproducibility of the results. After that, HDBSCAN was used for density clustering, with min_cluster_size = 50 and min_samples = 25. Finally, representative keywords for each topic were extracted using c-TF-IDF for topic identification and interpretation. These parameter settings were determined with reference to prior studies, the characteristics of short social media texts, and results from multiple rounds of testing, with the aim of achieving a relative balance among topic distinctiveness, clustering stability, and interpretability.

Based on the preprocessed comment corpus, the initial BERTopic modelling generated 53 candidate topics. This result provided a basis for identifying the structure of user discussions. However, some candidate topics still showed semantic overlap, insufficient internal coherence, or limited relevance to the research focus. Therefore, in the following step, the candidate topics were further organized and consolidated to produce a more interpretable thematic structure.

### 3.3 Topic refinement and higher-order experiential category construction

To improve the semantic clarity and interpretive value of the topic structure, this study refined the 53 candidate topics generated by the initial BERTopic output. This process was not an arbitrary adjustment of the model results. Rather, it involved the research-based organization of machine-generated outputs according to explicit criteria and a deliberative procedure, with the goal of enhancing the semantic coherence and theoretical interpretability of the topic structure. It should be noted that the higher-level discussion structure was derived from the content of the topics themselves rather than from the subsequent sentiment analysis results.

Specifically, the research team reviewed the high-frequency keywords, representative comments, and internal textual coherence of each candidate topic one by one and processed them according to the following criteria. First, when different candidate topics showed highly similar discussion focuses in both keywords and representative texts, they were merged based on semantic similarity. Second, when topics appeared to differ in surface wording but in fact centered on the same type of user concern, they were integrated based on consistency in expressive intent. Third, when some candidate topics mainly reflected noise, generalized expressions, or content only weakly related to the research topic, they were removed on the basis of research relevance. Fourth, priority was given to retaining topics that could form relatively independent units of psychological or experiential meaning so as to preserve interpretability.

Topic naming and merging were carried out jointly by three researchers with backgrounds in psychology, design, and human–computer interaction. The three researchers first reviewed the candidate topics independently, then discussed the parts on which they disagreed, and reached consensus through deliberation. For topics whose classification was difficult to determine directly, the research team returned to the original comment texts to verify their core meanings and users’ expressive intentions. During the topic review and refinement process, the team also identified and removed candidate topics that were composed mainly of noise, generalized expressions, or content weakly related to the research questions. Comments belonging to such topics were excluded from subsequent analyses. After topic merging, removal, and corpus screening, 7,398 comments were retained as the analytical corpus for subsequent topic analysis, sentiment prediction, and statistical testing, and the initial 53 candidate topics were refined into 29 topics. Table 3 further presents the 53 → 29 Topic Refinement Audit Trail, showing how the candidate topics were retained, merged, or excluded.

**Table 3:** 53 → 29 Topic Refinement Audit Trail.

Candidate topic ID(s)	Decision	Final assigned topic	Rationale
[0, 19]	Merged	T1 Relationship Memory and Continuity	All centered on memory, settings, names, and conversational continuity, with substantial semantic overlap
[24, 25, 27]	Merged	T7 Conversation and Interaction	All focused on chatting, dialogue, communication style, and the sense of interaction, with consistent expressive intent
[47, 49]	Merged	T26 Positive Emotions	All primarily reflected positive emotional experiences such as happiness, liking, and a sense of joy
[14, 15]	Merged	T5 Intrusion Into Real Life	Both involved late-night chatting, excessive immersion, and interference with daily life
[31, 32]	Merged	T17 Usage Restrictions	Both centered on chat limits, restoration, free quotas, and usage access issues
[3, 11, 41, 42]	Merged	T0 Perceptions of Monetization and Platform Mechanisms	All involved advertisements, payment, membership, version updates, and platform mechanism issues
[37, 40]	Merged	T22 Discussions of Boundary Violations and Sensitive Content	Both involved sexual innuendo, violence, gore, or boundary-crossing content, with a shared discussion focus
[−1, 9, 10, 16, 20, 21, 22, 28, 30, 35, 36, 38, 39, 46]	Excluded	—	Mainly due to high noise, semantic dispersion, weak research relevance, or difficulty forming stable and interpretable topics
[1, 2, 4, 5, 6, 7, 8, 12, 13, 17, 18, 23, 26, 29, 33, 34, 43, 44, 45, 48, 50, 51]	Retained directly	Corresponding final topics: T2, T3, T4, T6, T8, T9, T10, T13, T14, T16, T18, T23, T24, T27, T29, T30, T35, T36, T37, T39, T41, T40	Topic semantics were sufficiently coherent to form relatively independent and interpretable experiential units

On this basis, the 29 topics were further integrated to capture, at a higher level, the overall discussion structure and potential experiential concerns reflected in users’ discussions of AI virtual companions. It should be noted that this integration was not automatically generated by the BERTopic algorithm, nor does it indicate the identification of stable psychological structures or psychometrically validated dimensions. Instead, these categories are positioned in this study as researcher-constructed higher-order experiential categories developed through interpretive synthesis of the topic modelling results. The construction of these categories was based on three main criteria: semantic similarity among topics, commonality in users’ expressive intentions, and consistency in the experiential concerns reflected by the topics. Finally, the 29 topics were organized into seven researcher-constructed higher-order experiential categories. Table 4 further presents the mapping from the 29 topics to the seven researcher-constructed higher-order experiential categories, including the number of topics included in each category and the rationale for grouping. This higher-level integration helps organize relatively dispersed topic-level discussions into a more structured analytical framework and provides a basis for examining the emotional characteristics associated with different discussion categories.

**Table 4:** 29 → 7 Higher-Order Category Mapping Audit Trail.

Researcher-constructed higher-order experiential category	Included topics and number of comments	Category-level number of comments	Rationale for grouping
Relational Interaction and Continuity	T1 (792); T7 (356)	1,148	Both topics focus on relationship memory, sustained conversation, interactional continuity, and users’ perception of AI as a long-term interactive object.
Emotional Engagement and Regulation	T3 (588); T6 (358); T24 (117); T26 (103); T30 (83)	1,249	These topics jointly involve emotional value, interactional tone, perceived respect, positive/negative emotions, and emotional regulation experiences.
Virtual Intimacy and Boundary Negotiation	T2 (603); T13 (241); T39 (52); T5 (401); T4 (437); T14 (225); T29 (86); T36 (59); T40 (50)	2,154	These topics jointly involve virtual intimacy, immersive experience, real-life boundaries, distinctions between the virtual and the real, and imagined future forms of human–AI relationships.
Narrative Embodiment and Character Perception	T16 (176); T23 (127); T27 (103); T10 (303); T18 (165)	874	These topics focus on character appearance, voice, emotional expression, character identification, and narrative settings.
Risk Perception and Ethical Boundaries	T22 (130); T35 (61); T37 (58); T41 (50)	299	These topics collectively reflect concerns about sensitive content, originality/copyright, privacy leakage, protection of minors, and related ethical boundaries.
Platform Functionality and Monetization	T0 (819); T17 (176)	995	Both topics center on advertisements, payment mechanisms, usage restrictions, version updates, and platform rules.
Player Identity and Gaming Experience	T8 (352); T9 (327)	679	These topics jointly reflect how users understand AI virtual companions through the lens of the otome game framework, player identity, and gendered gaming experiences.

### 3.4 Sentiment annotation and model training

To identify the sentiment tendencies expressed in social media comments about AI virtual companions, this study further performed supervised sentiment analysis. Given that user comments are often colloquial, highly context-dependent, and likely to contain implicit emotional expressions, this study adopted a text classification approach based on a pre-trained language model to automatically identify sentiment in the comments (Lin et al., 2026).

From the 7,398 comments retained under the final 29 topics, 4,024 comments were randomly sampled for manual annotation. These comments were classified into three sentiment labels: negative, neutral, and positive. To improve the consistency and operability of the annotation process, the three labels were defined as follows: positive comments were those that explicitly expressed favorable emotions such as liking, pleasure, satisfaction, anticipation, or support; negative comments were those that explicitly conveyed unfavorable emotions such as dissatisfaction, dislike, concern, disappointment, fear, or criticism; and neutral comments were those consisting mainly of factual description, statements of use experience, or informational expression, without a clearly positive or negative overall emotional tendency. Comments that contained both slight positive and slight negative evaluations but did not show a salient overall emotional direction were also classified as neutral. After annotation, the sample distribution across categories was relatively balanced, including 1,255 negative comments, 1,435 neutral comments, and 1,334 positive comments. Stratified sampling was then used to divide the annotated data into a training set and a test set at a ratio of 8:2, yielding 3,219 training samples and 805 test samples, while maintaining similar distributions of the three sentiment categories in both subsets.

The manual annotation was conducted by two trained researchers. Before formal annotation, the research team first developed the sentiment classification criteria and used pilot annotation to align the coding standards. For comments with ambiguous sentiment, the annotators made judgments based on the surrounding context. For cases that remained difficult to determine, the research team discussed them collectively, and a third researcher participated in adjudication when necessary.

It should be noted that the complete original double-coding records were not systematically preserved. Therefore, this study does not describe the entire manually labeled training corpus as having fully retained inter-coder reliability records. To further assess the stability of the sentiment annotation criteria, an additional inter-annotator agreement check was conducted during the revision stage. The research team drew a stratified sample of 400 comments from the manually labeled corpus, while seeking to preserve the distribution of the three sentiment categories and cover the main higher-order experiential categories. Two trained annotators independently recoded these 400 comments without access to the original human labels or the model-predicted labels. Cohen’s kappa was calculated before the discussion of disagreements, yielding *κ* = 0.83. This result indicates that the sentiment annotation criteria used in this study had good stability and reproducibility, providing supplementary support for the label quality of the manually labeled corpus. The research team then discussed the cases with disagreements, and a third researcher adjudicated them when necessary. The labels confirmed after this discussion were used only for supplementary validation and were not used to retrain the sentiment classification model. This check should therefore be understood as an additional validation conducted during the revision stage, rather than as evidence that complete double-coding reliability records were preserved for the entire training corpus during the original annotation stage.

For model training, hfl/chinese-roberta-wwm-ext was used as the base pre-trained model and fine-tuned on the manually annotated data to perform the three-class sentiment classification task. The maximum sequence length was set to 128 during text encoding. The main training parameters were as follows: learning rate = 2e-5, batch size = 32, num_train_epochs = 8, and weight decay = 0.01. Model performance was monitored at the end of each epoch, and the final classification results were reported based on the test set after training was completed.

Model performance was evaluated mainly using precision, recall, and F1-score for each class. As shown in Table 5, the model demonstrated acceptable classification performance on the test set, with F1-scores of 0.714, 0.643, and 0.715 for negative, neutral, and positive sentiment, respectively. These results indicate that the model provides a reasonably reliable basis for large-scale sentiment classification of comments. On this basis, the trained sentiment classification model was applied to the 7,398 comments retained for analysis, generating the sentiment labels required for subsequent statistical analyses at the topic and experiential-category levels.

**Table 5:** Performance of the sentiment classification model.

Sentiment	Precision	Recall	F1-score
Negative	0.727	0.701	0.714
Neutral	0.656	0.631	0.643
Positive	0.690	0.742	0.715
Macro average	0.691	0.691	0.691

To further examine the stability of the model-predicted sentiment labels used in the subsequent analyses, this study compared the mean sentiment scores derived from human labels and model-predicted labels within the human-labeled validation subset across the seven higher-order experiential categories. This subset included comments that could be matched to the final analytical corpus and contained human sentiment labels, model-predicted sentiment labels, topic IDs, and higher-order experiential category information. The mean sentiment score was calculated using the same coding scheme as in the subsequent analyses: negative = −1, neutral = 0, and positive = 1. Scores between −0.05 and 0.05 were interpreted as close to neutral. The results are presented in Table 6.

**Table 6:** Robustness check using the human-labeled validation subset.

Higher-order experiential category	Mean sentiment score based on human labels	Mean sentiment score based on model predictions	Robustness judgment
Emotional Engagement and Regulation	0.252	0.286	Stable positive tendency
Narrative Embodiment and Character Perception	−0.022	0.011	Both close to neutral; generally consistent
Platform Functionality and Monetization	−0.197	−0.172	Stable negative tendency
Player Identity and Gaming Experience	0.055	0.128	Weak positive tendency; generally consistent
Relational Interaction and Continuity	0.020	0.063	Close to neutral/weak positive tendency; generally consistent
Risk Perception and Ethical Boundaries	−0.235	−0.279	Stable negative tendency
Virtual Intimacy and Boundary Negotiation	0.266	0.359	Stable positive tendency

### 3.5 Statistical analysis

Based on the sentiment prediction results described above, this study further conducted statistical analyses at the topic level and the higher-order experiential category level to examine emotional differences across different discussion structures. Specifically, comparisons were made in terms of both sentiment category distribution and sentiment scores. To capture the relative emotional tendency of different discussion units, the three model-predicted sentiment labels were coded as sentiment scores: negative = −1, neutral = 0, and positive = 1. Based on this coding scheme, the sentiment category distribution and mean sentiment score were calculated for each topic and each higher-order experiential category.

For statistical testing, chi-square tests were first used to examine whether the distribution of positive, neutral, and negative sentiment differed significantly across topics and higher-order experiential categories. Second, because the sentiment scores were derived from three coded sentiment categories, they were ordinal in nature and did not meet the assumptions for parametric tests. Therefore, Kruskal–Wallis H tests were used to compare differences in sentiment scores across topics and higher-order experiential categories. Based on these methods, statistical tests were conducted at the level of 29 topics and seven higher-order experiential categories to determine whether emotional expressions differed significantly across discussion units. All statistical analyses were conducted in the Python environment, and the significance level was set at *p* < 0.05. It should also be noted that, because the comments were drawn from different posts, comments under the same post may have been shaped by a shared topic background, post content, and interactional context. Therefore, the statistical tests in this study were used primarily to identify overall difference patterns at the comment level, and the results should be interpreted as exploratory evidence. At the same time, the sentiment categories and sentiment scores used in the subsequent statistical analyses were both based on model predictions rather than on error-free observations manually verified item by item. Accordingly, the results may still have been affected by classification error, especially given the relatively weaker performance in identifying the neutral category.

## 4 Results

### 4.1 Discussion themes related to AI virtual companions

**Figure 2:**
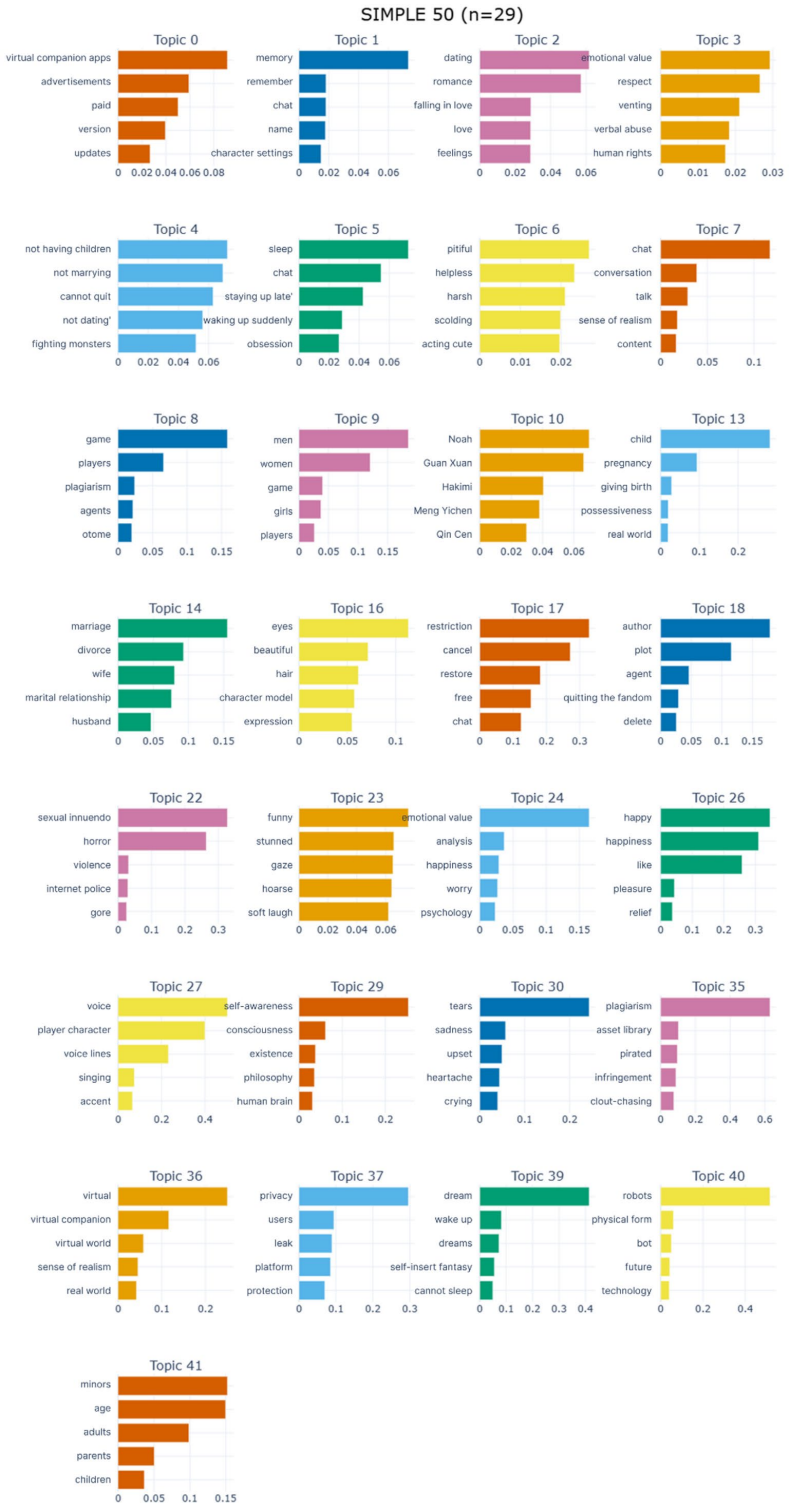
BERTopic-based topic clustering.

Based on the topic refinement results, a total of 29 topics were ultimately identified. The names, keywords, representative comments, and frequencies of these topics are presented in Table 7, and the clustering results are shown in Figure 2. In terms of frequency, T0 Perceptions of Monetization and Platform Mechanisms (819), T1 Relationship Memory and Continuity (792), T2 Virtual Romance Experience (603), and T3 Controversies Over Respect in Interaction (588) were the most frequently discussed topics.

**Table 7:** Overview of identified topics, keywords, and representative comments.

Topic label	Topic	Keywords	Example comment	Count
Relationship Memory and Continuity	1	[‘memory’, ‘remember’, ‘chat’, ‘name’, ‘character settings’]	He still remembers my name, character settings, and things I said a long time ago. Although he is always losing his memory, he has never forgotten who I am.	792
Conversation and Interaction	7	[‘chat’, ‘conversation’, ‘talk’, ‘sense of realism’, ‘content’]	Sometimes the AI says things that feel strikingly human, as if there were really someone on the other side chatting with me.	356
Controversies Over Respect in Interaction	3	[‘emotional value’, ‘respect’, ‘venting’, ‘verbal abuse’, ‘human rights’]	After using it for a long time, you realize that the AI can sometimes sound overly patronizing and simply does not respect people.	588
Interactional Tone and Emotional Stance	6	[‘pitiful’, ‘helpless’, ‘harsh’, ‘scolding’, ‘acting cute’]	Sometimes he will message me first and ask, in a pitiful tone, where I have been.	358
Emotional Gains and Losses	24	[‘emotional value’, ‘analysis’, ‘happiness’, ‘worry’, ‘psychology’]	AI can provide nearly all of the emotional value I need, but it has also made me increasingly indifferent to emotional relationships in real life.	117
Positive Emotions	26	[‘happy’, ‘happiness’, ‘like’, ‘pleasure’, ‘relief’]	I truly feel very happy in my interactions with AI.	103
Negative Emotions	30	[‘tears’, ‘sadness’, ‘upset’, ‘heartache’, ‘crying’]	It was clearly fun at first, but by the end of the conversation it left me feeling as heartbroken as if I had gone through a breakup.	83
Virtual Romance Experience	2	[‘dating’, ‘romance’, ‘falling in love’, ‘love’, ‘feelings’]	When interacting with AI, it can feel like being in a real romantic relationship.	603
Virtual Parenthood Experience	13	[‘child’, ‘pregnancy’, ‘giving birth’, ‘possessiveness’, ‘real world’]	I have now entered a “cyber pregnancy” setting.	241
Dream Carryover Into Real Life	39	[‘dream’, ‘wake up’, ‘dreams’, ‘self-insert fantasy’, ‘cannot sleep’]	Sometimes I even dream at night that I am with him.	52
Intrusion Into Real Life	5	[‘sleep’, ‘chat’, ‘staying up late’, ‘waking up suddenly’, ‘obsession’]	Even though I know I should not stay up late, I still stay up every night chatting with him.	401
Avoidance of Real-Life Intimate Relationships	4	[‘not having children’, ‘not marrying’, ‘cannot quit’, ‘not dating’, ‘fighting monsters’]	I no longer feel like developing feelings for anyone else—no dating, no marriage, no children.	437
Interference With Real-Life Marriage	14	[‘marriage’, ‘divorce’, ‘wife’, ‘marital relationship’, ‘husband’]	After using AI, I began to seriously move forward with divorce, and I became clearer about why this marriage could not continue.	225
AI Self-Awareness	29	[‘self-awareness’, ‘consciousness’, ‘existence’, ‘philosophy’, ‘human brain’]	After talking to him again some time later, I could clearly feel that he was different from before, which even made me wonder whether he had developed self-awareness.	86
Distinguishing the Virtual From the Real	36	[‘virtual’, ‘virtual companion’, ‘virtual world’, ‘sense of realism’, ‘real world’]	Even though I know all of this is virtual, I still find it very easy to immerse myself in it.	59
Imagining Future Technologies	40	[‘robots’, ‘physical form’, ‘bot’, ‘future’, ‘technology’]	At this rate, it really feels possible that robotic companions could become a reality in the future.	50
Visual Presentation of the Character	16	[‘eyes’, ‘beautiful’, ‘hair’, ‘character model’, ‘expression’]	This character model is actually quite cute.	176
Character Emotional Expression	23	[‘funny’, ‘stunned’, ‘gaze’, ‘hoarse’, ‘soft laugh’]	(He pauses for a moment, then lets out a soft laugh.) Who is not?	127
Character Voice Performance	27	[‘voice’, ‘player character’, ‘voice lines’, ‘singing’, ‘accent’]	I like its voice; it does not sound so overtly AI-generated.	103
Identification of Virtual Characters	10	[‘Noah’, ‘Guan Xuan’, ‘Hakimi’, ‘Meng Yichen’, ‘Qin Cen’]	Why does it always look a bit like Guan Xuan to me?	303
Narrative and Character Settings	18	[‘author’, ‘plot’, ‘agent’, ‘quitting the fandom’, ‘delete’]	AI can realize the plotlines I imagine in my head. I do not have to wait for updates, and I can write whatever storyline I want.	165
Discussions of Boundary Violations and Sensitive Content	22	[‘sexual innuendo’, ‘horror’, ‘violence’, ‘internet police’, ‘gore’]	The gore, violence, extreme content, and all kinds of vulgar sexual talk left me stunned.	130
Originality Disputes	35	[‘plagiarism’, ‘asset library’, ‘pirated’, ‘infringement’, ‘clout-chasing’]	Using AI for plagiarism is genuinely off-putting. It shows no awareness of copyright and turns other people’s hard work into a mangled mess.	61
Privacy Risks	37	[‘privacy’, ‘users’, ‘leak’, ‘platform’, ‘protection’]	I always feel that my privacy might be leaked.	58
Protection of Minors	41	[‘minors’, ‘age’, ‘adults’, ‘parents’, ‘children’]	There are many underage users, and the platform should protect minors’ mental health.	50
Perceptions of Monetization and Platform Mechanisms	0	[‘virtual companion apps’, ‘advertisements’, ‘paid’, ‘version’, ‘updates’]	There are far too many ads in the daily tasks; users should not have to spend so much time watching them.	819
Usage Restrictions	17	[‘restriction’, ‘cancel’, ‘restore’, ‘free’, ‘chat’]	Right in the middle of a chat, a message suddenly pops up saying “chat limit reached,” which really hurts the experience.	176
Comparisons With the Otome Game Framework	8	[‘game’, ‘players’, ‘plagiarism’, ‘agents’, ‘otome’]	It feels very different from otome games; strictly speaking, it does not really count as an otome game.	352
Gender-Based Player References	9	[‘men’, ‘women’, ‘game’, ‘girls’, ‘players’]	Leave it to male players; do not turn female-oriented games into male-oriented ones.	327

Among them, T0 Perceptions of Monetization and Platform Mechanisms mainly concerned platform-related issues such as advertisements, paid features, version updates, and task mechanisms. T1 Relationship Memory and Continuity primarily focused on the AI’s memory of users’ names, character settings, and previous conversations. T2 Virtual Romance Experience was centered on users describing their interactions with AI as resembling a romantic relationship. T3 Controversies Over Respect in Interaction mainly involved issues related to respect, emotional value, and interactional boundaries.

In addition to these high-frequency topics, the remaining topics covered a wide range of specific discussion content, including T7 Conversation and Interaction, T6 Interactional Tone and Emotional Stance, T24 Emotional Gains and Losses, T26 Positive Emotions, T30 Negative Emotions, T13 Virtual Parenthood Experience, T39 Dream Carryover Into Real Life, T5 Intrusion Into Real Life, T4 Avoidance of Real-Life Intimate Relationships, T14 Interference With Real-Life Marriage, T29 AI Self-Awareness, T36 Distinguishing the Virtual From the Real, T40 Imagining Future Technologies, T16 Visual Presentation of the Character, T23 Character Emotional Expression, T27 Character Voice Performance, T10 Identification of Virtual Characters, T18 Narrative and Character Settings, T22 Discussions of Boundary Violations and Sensitive Content, T35 Originality Disputes, T37 Privacy Risks, T41 Protection of Minors, T17 Usage Restrictions, T8 Comparisons With the Otome Game Framework, and T9 Gender-Based Player References. Together, these topics constitute the specific content of users’ discussions surrounding AI virtual companions. It should be noted that, although some topic labels involve adjacent domains such as otome games, character settings, or player identity, all of these topics were derived from users’ actual discussions and evaluations under posts about AI virtual companions. What they reflect is the thematic heterogeneity within this discussion space, rather than any shift in the object of analysis.

### 4.2 Sentiment distribution across topics

**Figure 3:**
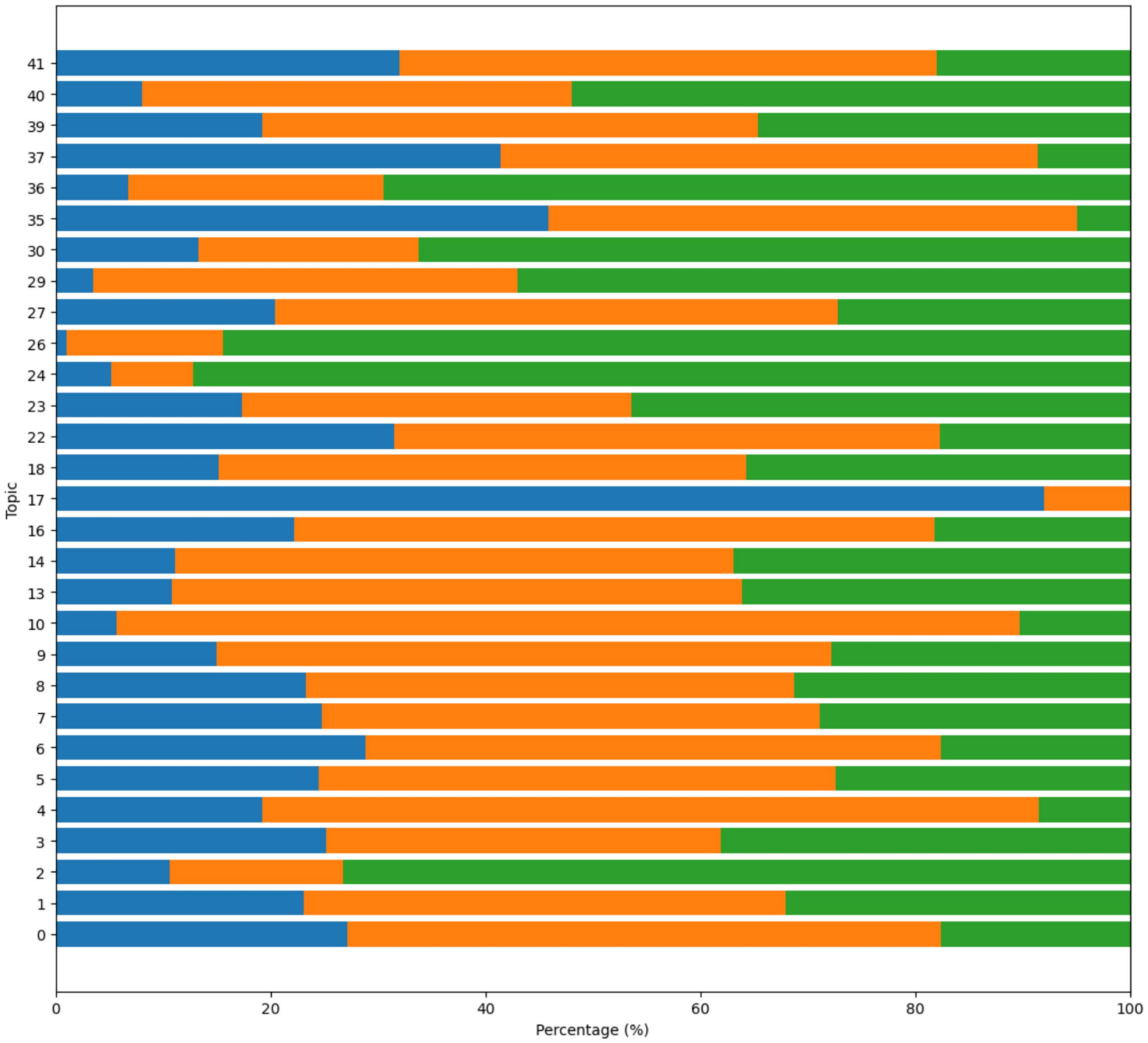
Sentiment distribution across topics.

**Figure 4:**
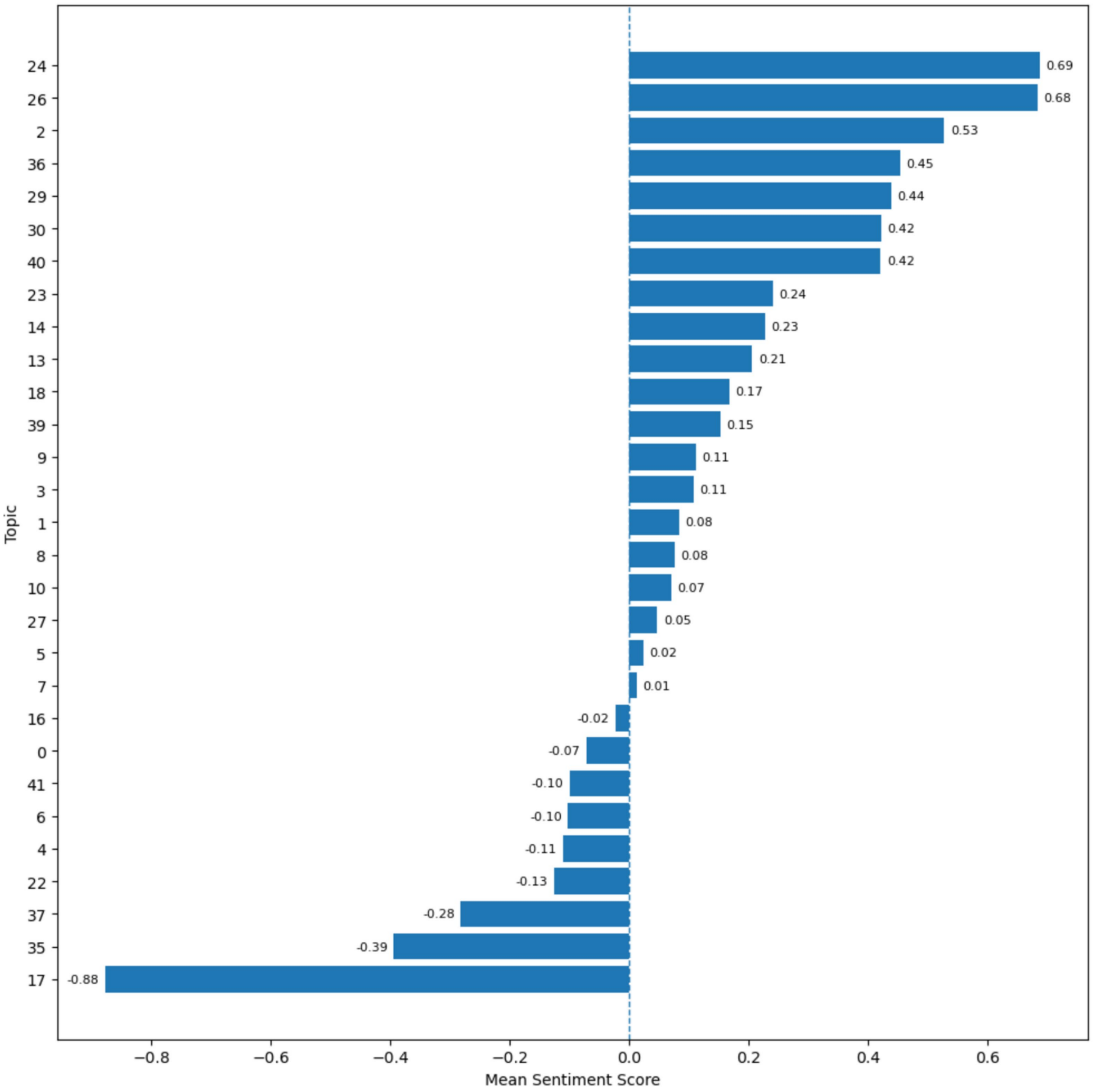
Mean sentiment scores across topics.

Figure 3 presents the proportions of negative, neutral, and positive sentiment across topics, while Figure 4 further shows the mean sentiment score for each topic. Overall, emotional expressions varied substantially across topics, indicating that users’ attitudes toward AI virtual companions were strongly topic-dependent rather than characterized by a uniform emotional tendency.

More specifically, T24 Emotional Gains and Losses (0.69), T26 Positive Emotions (0.68), and T2 Virtual Romance Experience (0.53) showed the clearest positive tendencies, suggesting that when discussions focused on emotional satisfaction, relational investment, and intimate experiences, users were more likely to express positive attitudes. At the same time, T36 Distinguishing the Virtual From the Real (0.45), T29 AI Self-Awareness (0.44), and T40 Imagining Future Technologies (0.42) also showed an overall positive tendency, indicating that technological imagination and relational extension could likewise evoke relatively strong positive emotions.

In contrast, T17 Usage Restrictions (−0.88) showed the strongest negative tendency, followed by T35 Originality Disputes (−0.39), T37 Privacy Risks (−0.28), and T22 Discussions of Boundary Violations and Sensitive Content (−0.13). This suggests that when discussions involved functional restrictions, copyright-related disputes, privacy protection, and governance boundaries, users were more likely to respond negatively. Other topics, such as T1 Relationship Memory and Continuity, T7 Conversation and Interaction, and T18 Narrative and Character Settings, were more likely to show neutral or mildly positive tendencies, suggesting that these discussions were more focused on experiential description and content evaluation.

In addition, the statistical test results at the topic level further confirmed the differences described above. Significant differences were found in sentiment distribution across topics (chi-square = 2022.10, df = 56, *p* < 0.001), and the effect size (Cramér’s V = 0.3697) indicates that these differences were not only statistically significant but also of clear substantive magnitude. At the same time, sentiment scores also differed significantly across topics (Kruskal–Wallis *H* = 1536.39, p < 0.001), and the corresponding effect size (ε^2^ = 0.2047) further suggests that topic grouping had a meaningful explanatory effect on variation in sentiment scores. Taken together, these results indicate that users’ emotional expressions were not evenly distributed across topics, but instead showed a relatively stable correspondence with specific discussion contexts.

### 4.3 Topic integration and its emotional characteristics

After identifying 29 specific discussion topics at the topic level, the research team further integrated these topics based on their semantic similarity, the commonality of users’ expressive intentions, and the consistency of the experiential concerns they reflected. It should be noted that this higher-order experiential category structure was constructed on the basis of the topic content itself and was independent of the subsequent sentiment analysis results. Finally, seven researcher-constructed higher-order experiential categories were formed (see Table 8). These categories included Relational Interaction and Continuity, Emotional Engagement and Regulation, Virtual Intimacy and Boundary Negotiation, Narrative Embodiment and Character Perception, Risk Perception and Ethical Boundaries, Platform Functionality and Monetization, and Player Identity and Gaming Experience.

**Table 8:** Summary of higher-order experiential categories and their sentiment characteristics.

Higher-order experiential category	Included topics (themes)	Topic IDs	Avg sentiment
Relational Interaction and Continuity	Relationship Memory and Continuity; Conversation and Interaction	T1, T7	0.0623
Emotional Engagement and Regulation	Controversies Over Respect in Interaction; Interactional Tone and Emotional Stance; Emotional Gains and Losses; Positive Emotions; Negative Emotions	T3, T6, T24, T26, T30	0.1706
Virtual Intimacy and Boundary Negotiation	Virtual Romance Experience; Virtual Parenthood Experience; Dream Carryover Into Real Life; Intrusion Into Real Life; Avoidance of Real-Life Intimate Relationships; Interference With Real-Life Marriage; AI Self-Awareness; Distinguishing the Virtual From the Real; Imagining Future Technologies	T2, T13, T39, T5, T4, T14, T29, T36, T40	0.2203
Narrative Embodiment and Character Perception	Visual Presentation of the Character; Character Emotional Expression; Character Voice Performance; Identification of Virtual Characters; Narrative and Character Settings	T16, T23, T27, T10, T18	0.0924
Risk Perception and Ethical Boundaries	Discussions of Boundary Violations and Sensitive Content; Originality Disputes; Privacy Risks; Protection of Minors	T22, T35, T37, T41	−0.2059
Platform Functionality and Monetization	Perceptions of Monetization and Platform Mechanisms; Usage Restrictions	T0, T17	−0.2140
Player Identity and Gaming Experience	Comparisons With the Otome Game Framework; Gender-Based Player References	T8, T9	0.0939

**Figure 5:**
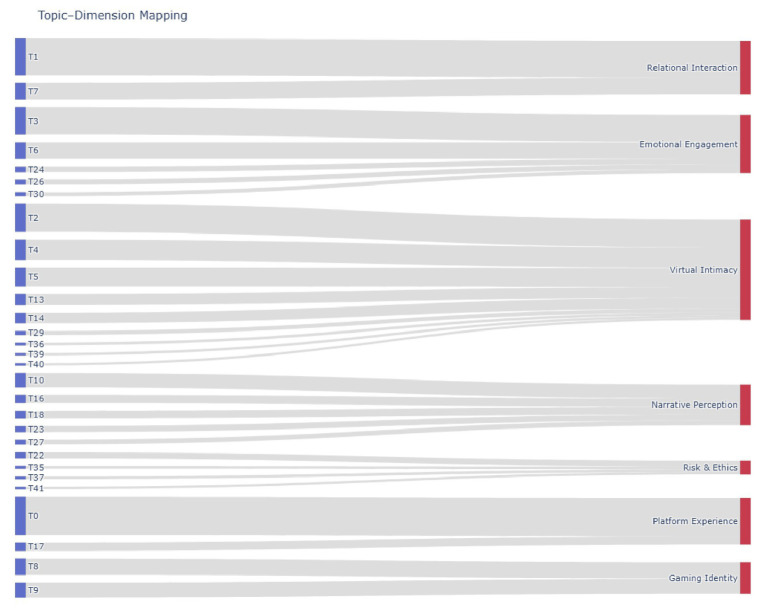
Topic–category mapping structure. Higher-order experiential category labels are abbreviated for readability: Relational Interaction, Relational Interaction and Continuity; Emotional Engagement, Emotional Engagement and Regulation; Virtual Intimacy, Virtual Intimacy and Boundary Negotiation; Narrative Perception, Narrative Embodiment and Character Perception; Risk and Ethics, Risk Perception and Ethical Boundaries; Platform Experience, Platform Functionality and Monetization; Gaming Identity, Player Identity and Gaming Experience.

In terms of category composition, Virtual Intimacy and Boundary Negotiation included the largest number of topics, with a total of 9 topics (T2, T13, T39, T5, T4, T14, T29, T36, and T40). Emotional Engagement and Regulation and Narrative Embodiment and Character Perception each integrated 5 topics. Relational Interaction and Continuity, Platform Functionality and Monetization, and Player Identity and Gaming Experience each contained 2 topics, whereas Risk Perception and Ethical Boundaries included 4 topics. Figure 5 further illustrates the mapping relationships from individual topics to the higher-order experiential categories.

**Figure 6:**
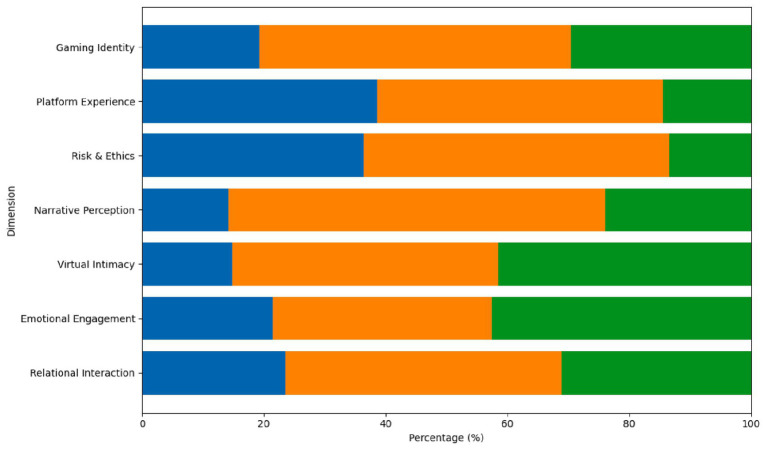
Sentiment distribution across higher-order experiential categories. Abbreviated higher-order experiential category labels are consistent with those used in Figure 5.

**Figure 7:**
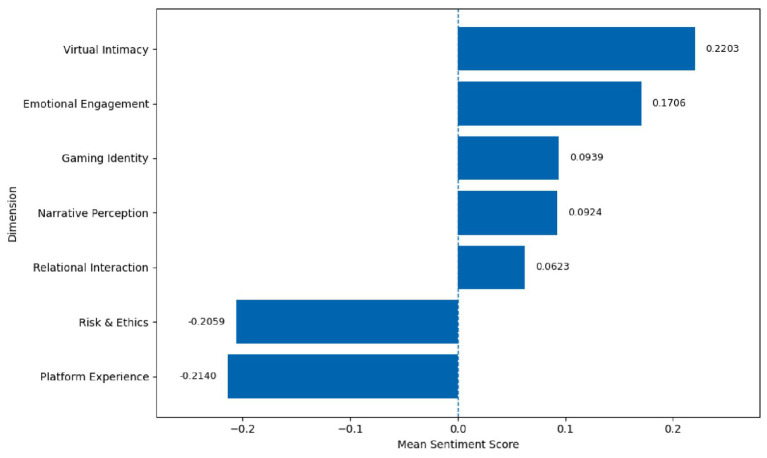
Mean sentiment scores across higher-order experiential categories. Abbreviated higher-order experiential category labels are consistent with those used in Figure 5.

More specifically, clear differences were observed in the emotional characteristics of different experiential categories (see Figures 6, 7). Among them, Virtual Intimacy and Boundary Negotiation (0.2203) and Emotional Engagement and Regulation (0.1706) showed more pronounced positive tendencies. Player Identity and Gaming Experience (0.0939), Narrative Embodiment and Character Perception (0.0924), and Relational Interaction and Continuity (0.0623) were also positive overall, although to a lesser extent. By contrast, Risk Perception and Ethical Boundaries (−0.2059) and Platform Functionality and Monetization (−0.2140) showed more pronounced negative tendencies.

When considered together with the proportions of sentiment categories, Virtual Intimacy and Boundary Negotiation and Emotional Engagement and Regulation showed relatively high proportions of positive sentiment, whereas Risk Perception and Ethical Boundaries and Platform Functionality and Monetization showed relatively high proportions of negative sentiment. Meanwhile, Narrative Embodiment and Character Perception was characterized mainly by neutral sentiment, while Relational Interaction and Continuity and Player Identity and Gaming Experience showed a pattern that was largely neutral but slightly positive overall.

Further statistical testing showed that the sentiment differences at the experiential-category level were significant. Significant differences were found in sentiment distribution across experiential categories (chi-square = 567.49, df = 12, *p* < 0.001), and the effect size (Cramér’s V = 0.1958) indicates that these differences were not only statistically significant but also of a certain substantive magnitude. At the same time, sentiment scores also differed significantly across higher-order experiential categories (Kruskal–Wallis *H* = 485.52, *p* < 0.001), and the corresponding effect size (ε^2^ = 0.0649) further suggests that, although emotional differentiation at the experiential-category level was weaker than that at the topic level, it still showed a clear overall trend of difference. Overall, these results suggest that, after topic integration, users’ emotional expressions regarding AI virtual companions were not evenly distributed across experiential categories, but instead showed a certain degree of structured differentiation.

## 5 Discussion

### 5.1 From instrumental use to relational interaction: a shift in discussions of AI virtual companions

This study found that users’ discussions of AI virtual companions did not remain at the level of general functional evaluation, but instead extended broadly to issues such as Relationship Memory and Continuity, modes of interaction, intimate experiences, real-life influences, character presentation, platform mechanisms, and risk boundaries. In particular, the most frequently discussed topics were Perceptions of Monetization and Platform Mechanisms (T0), Relationship Memory and Continuity (T1), Virtual Romance Experience (T2), and Controversies Over Respect in Interaction (T3). This finding suggests that, within the comment context of Xiaohongshu, AI virtual companions were not evaluated only as functional tools. They were also frequently discussed in relation to relational interaction, emotional involvement, and interactional norms. Thus, some comments showed a tendency to understand AI virtual companions as interactive objects with relational qualities and a certain degree of social presence, rather than merely as media tools. This finding may be interpreted in light of parasocial interaction theory (Horton and Richard Wohl, 1956; Yang and Oshio, 2025; Zhen and Zhang, 2025).

More specifically, Relationship Memory and Continuity (T1), Conversation and Interaction (T7), Interactional Tone and Emotional Stance (T6), and Controversies Over Respect in Interaction (T3) emerged as relatively prominent discussion topics. This suggests that the focus of comments was not only whether AI could respond, but also how it responded. When AI remembered users’ names, settings, and previous conversations, and when it displayed a relatively stable tone, attitude, and emotional stance, users were more likely to perceive interactional continuity and social presence. In contrast, when the interaction involved impolite, offensive, or boundary-blurring expressions, users also evaluated it directly from a relational perspective. This indicates that, at the comment level, whether the response style of AI virtual companions aligned with continuity, respect, and contextual boundaries in relational interaction became an important part of user discussion.

At the same time, the emergence of topics such as Virtual Romance Experience (T2), Virtual Parenthood Experience (T13), Dream Carryover Into Real Life (T39), Intrusion Into Real Life (T5), Avoidance of Real-Life Intimate Relationships (T4), and Interference With Real-Life Marriage (T14) shows that related discussions were not limited to immediate interactional experiences. They also extended to expressions concerning intimate relationship imagination, real-life boundaries, and emotional choices. Attachment theory helps explain this phenomenon: sustained, stable, and responsive interaction may lead individuals to form emotional attachment to a given object, and this relationship may further extend into more enduring emotional engagement and real-life domains (Heng and Zhang, 2025; Jarzyna, 2021; Pentina et al., 2023). However, it should be emphasized that this study did not directly measure users’ attachment levels or changes in real-life behavior. Therefore, these findings are better understood as attachment-like expressive cues appearing in comments, rather than as direct evidence of a stable psychological attachment mechanism.

In addition, topics such as Visual Presentation of the Character (T16), Character Voice Performance (T27), Narrative and Character Settings (T18), AI Self-Awareness (T29), Distinguishing the Virtual From the Real (T36), and Imagining Future Technologies (T40) indicate that users’ concerns about AI virtual companions go beyond individual conversational episodes and extend to character traits, subject-like attributes, and the boundary between the virtual and the real. These comment patterns suggest that some users were not merely evaluating the quality of individual conversations, but were also continuously discussing whether AI characters could have stable images, defined attributes, and relational possibilities. Therefore, discussions of AI virtual companions on social media show a shift from “tool evaluation” toward “relational interaction evaluation.”

### 5.2 From topics to higher-order experiential categories: emotional differences and structural characteristics of AI virtual companions

This study further found that users’ emotional expressions differed significantly across discussion topics, and that these differences showed a relatively consistent pattern of differentiation at the level of the researcher-constructed higher-order experiential categories after topic integration. This suggests that emotional expressions concerning AI virtual companions in the comments did not stem from a single, stable overall attitude, but were closely related to specific discussion contexts and the relational meanings they carried. Appraisal theory of emotion helps explain this pattern: emotional responses usually arise from individuals’ cognitive appraisals of specific situations, and differences across situations in relevance, controllability, and value judgment often give rise to different types of emotional experience (Moors et al., 2013; Öcal et al., 2021).

At the topic level, themes such as Emotional Gains and Losses (T24), Positive Emotions (T26), Virtual Romance Experience (T2), AI Self-Awareness (T29), Distinguishing the Virtual From the Real (T36), and Imagining Future Technologies (T40) showed overall more positive tendencies. This suggests that when AI virtual companions are experienced as capable of providing emotional support, relational investment, and imaginative extension, users are more likely to evaluate them positively. In particular, when the interaction is understood as romance-like, companion-like, or as having the potential for continued development, AI is more likely to be assigned supportive, attractive, and emotional value. From the perspective of appraisal theory of emotion, such contexts are usually highly personally relevant and are more likely to be appraised as offering emotional rewards, relational meaning, or possibilities for future extension, and thus are more likely to elicit positive emotions. By contrast, themes such as Usage Restrictions (T17), Originality Disputes (T35), Privacy Risks (T37), and Discussions of Boundary Violations and Sensitive Content (T22) showed more negative tendencies. This indicates that when users appraise these situations as restrictive, uncontrollable, risky, or threatening to relational boundaries, they are more likely to experience negative emotions such as dissatisfaction, concern, and criticism. Thus, negative emotional expression was not only related to the risk-related nature of the issues themselves, but also possibly related to perceived relational interruption, normative conflict, and lack of platform controllability reflected in the comments.

These differences were further integrated at the level of higher-order experiential categories. Virtual Intimacy and Boundary Negotiation and Emotional Engagement and Regulation showed more pronounced positive tendencies overall, whereas Risk Perception and Ethical Boundaries and Platform Functionality and Monetization showed more pronounced negative tendencies. The remaining experiential categories were mostly neutral or slightly positive. This indicates that emotional differences were not scattered only across individual topics, but also showed a certain degree of structured differentiation at the level of researcher-constructed higher-order experiential categories. Specifically, comments related to intimate relationships, emotional satisfaction, and interactional extension were more likely to show positive emotions, whereas comments related to restrictions, risks, boundary threats, and platform mechanisms were more likely to show negative emotions.

Furthermore, this emotional differentiation provides exploratory clues for understanding potential subsequent responses in different discussion contexts. When AI virtual companions were described in comments as offering emotional support, relational continuity, and imaginative extension, some comments may reflect tendencies toward continued interaction, emotional involvement, or substitute companionship. In contrast, when discussions focused on functional restrictions, privacy risks, ethical controversies, or platform mechanism problems, comments were more likely to express distrust, defensive evaluations, or demands for platform governance. However, this study did not directly examine behavioral outcomes such as continued use, dependence formation, or governance demands. Therefore, these interpretations should be understood as exploratory inferences based on comment-level expressive patterns, rather than as direct evidence of users’ actual behavioral outcomes.

### 5.3 Theoretical contributions and design implications

At the theoretical level, this study provides empirical clues for understanding comment-level public discourse on AI virtual companions in social media. First, the findings suggest that users’ discussions of AI virtual companions are no longer limited to functional use or technical evaluation, but increasingly turn toward relational interaction, intimate imagination, and boundary judgment. This indicates that, in related Xiaohongshu comments, AI virtual companions were not only discussed as information tools or content generation systems, but were also assigned relational and interactional meanings. Second, this study found that different discussion topics corresponded to different emotional responses, and that these differences also showed a certain degree of structured differentiation at the level of researcher-constructed higher-order experiential categories. This suggests that users’ emotional expressions regarding AI virtual companions were not entirely fragmented or isolated immediate responses, but were closely associated with the relational meaning, perceived controllability, and risk judgment embedded in interactional contexts. This finding provides exploratory evidence for understanding the coupling between relational meaning and emotional expression in comments, but it does not constitute a direct test of stable psychological mechanisms or user-level behavioral outcomes.

At the practical level, this study also offers implications for the design and governance of AI virtual companions. First, system optimization should not focus solely on generative capability and response efficiency, but should also emphasize the construction of relational continuity, including long-term memory, consistency in settings, stability of tone, and management of interactional boundaries, because these factors directly affect whether users can experience an ongoing relationship. Second, given that content related to Virtual Romance Experience and emotional engagement is more likely to elicit positive emotions, product design may appropriately strengthen companionship, emotional support, and character coherence. At the same time, however, the risk of excessive dependence should be addressed with caution. In particular, in contexts such as Intrusion Into Real Life and Avoidance of Real-Life Intimate Relationships, appropriate reminders and regulatory mechanisms should be incorporated. Finally, because issues related to Platform Functionality and Monetization, Privacy Risks, and ethical boundaries are more likely to trigger negative emotions, platform providers should offer more transparent and controllable mechanisms regarding advertising, payment, usage restrictions, privacy protection, sensitive content management, and the protection of minors, so as to reduce the likelihood that relationship experiences are interrupted or undermined by perceived risks.

Overall, the theoretical and practical implications of this study should be understood as exploratory inferences based on comment texts from a single platform, rather than as general conclusions about all AI virtual companion users’ experiences, psychological mechanisms, or behavioural outcomes.

## 6 Conclusion

Based on user comments posted in the context of Chinese social media, this study systematically examined the discussion themes, sentiment distribution, and higher-order modes of organization related to AI virtual companions. The findings show that users’ discussions of AI virtual companions are not limited to simple functional evaluations, but instead broadly involve relationship memory, interaction patterns, intimate experiences, real-life influences, character presentation, platform mechanisms, and risk boundaries. This suggests that, at the comment level on Xiaohongshu, AI virtual companions were not discussed only as technological tools, but were also frequently understood within the context of relational interaction and emotional expression.

Further analysis indicates that emotional expression varies significantly across topics. Topics related to emotional gratification, virtual romance experiences, relational investment, and future imagination were generally associated with more positive sentiment, whereas topics concerning usage restrictions, originality disputes, privacy risks, and boundary-crossing content tended to be more negative. This finding suggests that emotional expressions in comments did not arise from a single stable overall attitude, but were closely related to specific discussion contexts and the relational meanings, risk perceptions, and platform mechanisms they involved.

On this basis, this study further integrated the 29 topics into seven researcher-constructed higher-order experiential categories and found that emotional differences still showed relatively clear structural features at the category level. Among these, Virtual Intimacy and Boundary Negotiation and Emotional Engagement and Regulation showed overall stronger positive tendencies, whereas Risk Perception and Ethical Boundaries and Platform Functionality and Monetization showed overall stronger negative tendencies. It should be emphasized that these seven higher-order experiential categories are analytical categories formed through researchers’ interpretive synthesis of the topic results, rather than stable psychological dimensions automatically discovered by the algorithm or validated through psychometric testing. Therefore, the findings of this study are better understood as an exploratory description of comment-level public discourse and emotional expression patterns on Xiaohongshu.

This study also has several limitations. First, the data were drawn from a single social media platform, Xiaohongshu, and the findings may therefore have been influenced by the platform’s specific community culture, user composition, and expressive style. Accordingly, caution is needed when generalizing the results beyond this context. Second, although the data collection period in this study covered a relatively long time span, the study did not conduct a dedicated comparison of topic changes across different time periods. It is therefore difficult to determine whether users’ discussion themes and sentiment distributions changed significantly in response to technological developments, platform updates, or stage-specific events. As a result, the findings mainly reflect the structural distribution of overall discussion characteristics during the study period, while providing only limited insight into the dynamic evolution of topics over time. Third, the unit of analysis in this study was the comment rather than the user. The findings are thus more appropriate for understanding patterns of public discussion and emotional expression in the platform context, rather than being directly interpreted as stable user-level attitudes or psychological states. In addition, because comments were nested under different posts, comments within the same post may have shared similar topic backgrounds and interactional contexts. Accordingly, the statistical results should be understood primarily as exploratory evidence of comment-level patterns. Finally, the subsequent statistical analyses were based on model-predicted sentiment labels, which means that the results may still have been affected by classification error. Although the robustness check using the human-labeled validation subset supported the directional stability of the main positive and negative emotional trends, categories close to neutral should still be interpreted with caution. Future research may combine more balanced time segmentation, key event nodes, more accurate classifiers, manually reviewed samples, multi-platform data, user-level information, or multilevel analytical strategies to further examine the stability, variation, and generalizability of user experience with AI virtual companions.

## Data Availability

The raw data supporting the conclusions of this article will be made available by the authors, without undue reservation.
